# Tissue engineering to better understand senescence: Organotypics come of age

**DOI:** 10.1016/j.mad.2020.111261

**Published:** 2020-09

**Authors:** Deborah A. Milligan, Eleanor J. Tyler, Cleo L. Bishop

**Affiliations:** Blizard Institute of Cell and Molecular Science, Barts and The London School of Medicine and Dentistry, 4 Newark Street, London E1 2AT, UK

**Keywords:** Tissue engineering, 3D organotypic culture, Senescence, Living skin equivalent, Remodelling the microenvironment, Drug discovery and development

## Abstract

The recent advent of ‘organs in a dish’ has revolutionised the research landscape. These 3D culture systems have paved the way for translational, post genomics research by enabling scientists to model diseases in the laboratory, grow patient-derived organoids, and unite this technology with other cutting-edge methodologies such as drug discovery. Fields such as dermatology and neuroscience have revolutionised the development of robust 3D models, which faithfully recapitulate native physiology *in vivo* to provide important functional and mechanistic insights. These models have underpinned a rapid growth in the number of organs and myriad of human diseases that can be modelled in 3D, which currently includes breast, cerebral cortex, heart, intestine, kidney, liver, lung, neural tube, pancreas, prostate, skin and stomach, as well as patient derived tumours. However, so far, they have not yet been employed extensively in the study of fundamental cellular programmes such as senescence. Thus, tissue engineering and 3D culture offer an exciting opportunity to further understand the bright and dark sides of senescence in a more complex and physiologically relevant environment. Below, we will discuss previous approaches to investigating senescence and ageing using organotypic models, and some potential opportunities for future research.

## 1 Methodologies of 3D organotypic culture

In recent years, it has become clear that senescence is important in a myriad of biological processes, which can be both beneficial and detrimental. Senescence prevents tumourigenesis (Serrano et al., 1997), facilitates wound healing (Demaria et al., 2014), promotes normal embryonic development (Munoz-Espin et al., 2013; Storer et al., 2013), and stimulates regeneration (Mosteiro et al., 2018; Yun et al., 2015), to name a few important beneficial roles of the ‘bright side’ of senescence. By contrast, senescence can also be detrimental, with the ‘dark side’ of the senescence-associated secretory phenotype (SASP) spreading paracrine senescence and inducing chronic inflammation within the microenvironment, thus contributing to ageing and age-related disease (Acosta et al., 2013; Coppe et al., 2008). We suggest that 3D organotypic modelling provides an opportunity to better understand senescent cells in both their favourable and deleterious contexts, enabling us to develop novel therapeutic strategies exploiting the bright and dark sides of senescence.

**Fig. 1:**
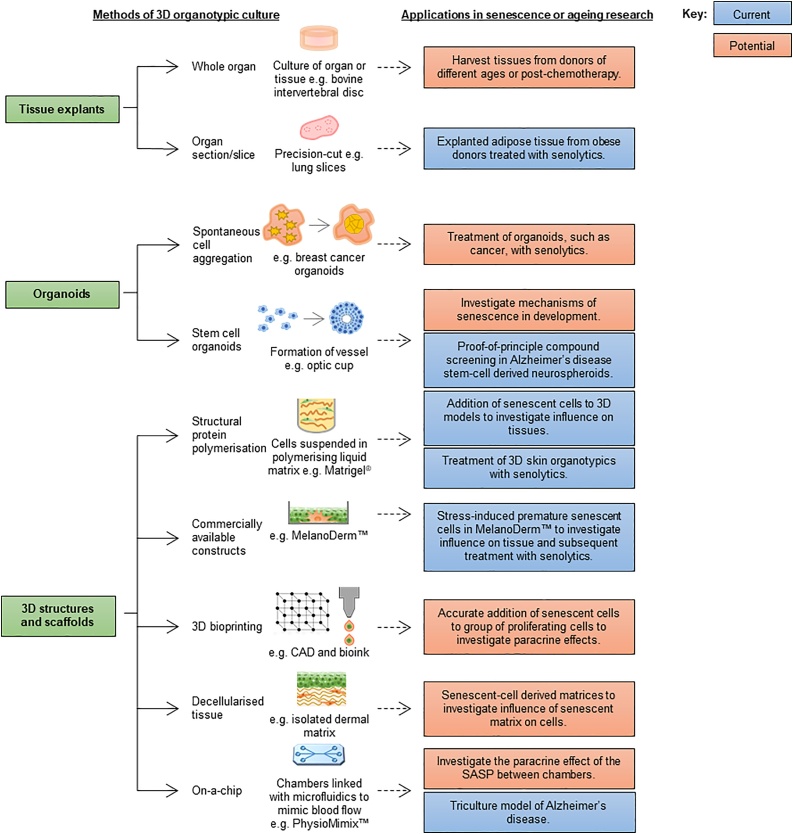
**Overview of current methodologies of 3D organotypic culture and future applications for organotypics in senescence research.** Methods of organotypic culture are divided into 3 categories (left): tissue explants, organoids and 3D structures and scaffolds. Tissue explants include the use of whole organ culture and organ sections or slices. Organoids can be sub-divided into spontaneously forming aggregates or inducible stem cell organoids. Finally, 3D structures include the use of structural protein polymerisation, commercially available constructs, models built using 3D bioprinting techniques, decellularised human or animal model tissue and ‘on-a-chip’ assays. Examples of current applications of these techniques are shown in blue, whereas opportunities for future applications are shown in orange.

To date, a spectrum of different approaches have been established for organotypic modelling including: spontaneous formation of 3D structures using cell suspension; re-suspension of cells within a 3D scaffold matrix; 3D cell printing; and ‘on-a-chip’ microfluidics (summarised in Fig. 1). In some cases, organ explants are also considered to be a form of organotypic modelling. One reason for the success of organotypic models is that these 3D assays have a number of advantages over traditional 2D on-a-plastic methods. These include, but are not limited to, the presence of defined or tuneable extracellular matrix (ECM), the potential to include multiple cell types, and the opportunity to differentiate cells in response to spatiotemporal cues. Several groups have established that there are important morphological, biochemical and functional differences between cells cultured in 2D *versus* 3D (Kenny et al., 2007; Sung et al., 2013; Shichi et al., 2019), suggesting there is scope to better understand native cellular behaviour using 3D assays.

The different methods of tissue engineering and 3D organotypic culture that are currently in use are summarised in Fig. 1. A number of tissues, such as skin, can be isolated directly from an animal model or human donor and remain viable *in vitro* for several weeks. Such *ex vivo* cultures are a useful tool to study the effects of a treatment or other influencing factor on a given tissue. Whole organ explants, such as intervertebral discs (Pfannkuche et al., 2019), or organ slices, such as lung (Liu et al., 2019) have been utilised for this purpose. Organoids, which are organ-like structures formed from cell suspensions, can be used to study the mechanisms by which tissues organise themselves and can be a more economical use of cells. For example, breast cancer cells are able to spontaneously aggregate in culture to create mammary organoids, which can then be useful to test clinically relevant compounds (Kenny et al., 2007). Furthermore, through the use of growth factors, stem cells can recapitulate intrinsic self-organising and differentiation programmes to form embryonic stem (ES) cell derived optic cups (Eiraku et al., 2011). This approach has allowed a greater understanding of stem cell behaviour and, in the future, may also provide the opportunity to manipulate development for the purposes of tissue regeneration.

Creating a scaffold on which cells can grow is another widely used method. A number of different techniques are used to achieve this type of assay, for example suspending cells in a polymer matrix that undergoes gelation at 37 °C, thus creating a solid matrix (Benton et al., 2014). Alternatively, tissue extracted from animal models can be decellularised to create a physiologically relevant matrix that can be repopulated with cells of interest (Wu et al., 2018). Commercially available alternatives to manually create matrices are now widely available, including empty matrices that can be populated with cells and tissue models containing mixed populations for investigations in 3D. Furthermore, computer aided design (CAD) and 3D bioprinting has allowed recapitulation of premeditated tissue models, facilitating the investigation of specific spatial relationships with a high level of consistency (Huang et al., 2017). Finally, the emergence of microfluidic technologies has led to the development of ‘on-a-chip’ models, which can represent an organ, or multiple organs, within chambers on a slide, linked with microfluidic capillaries to represent blood flow (Huang et al., 2017). Such models are proving useful to further our understanding of the effects of migration and cellular communication in 3D contexts (Park et al., 2018).

## 2 Applications for organotypics in senescence research

The field of senescence predominately uses 2D tissue culture and/or *in vivo* animal models, but there are a number of ways in which 3D culture could be applied to ageing and senescence studies to add to our growing knowledge of these processes. The opportunity these models present is beginning to emerge, and some of the ways in which they have been utilised are discussed below.

### 2.1 Cellular crosstalk and paracrine signalling

Co-culture of multiple cell types allows for a better understanding of signalling crosstalk, which facilitates the efficient function of tissues and organs. However, in 2D, the true nature of cellular communication may be masked by the lack of ECM and changes in cell-cell contact. By constructing a more physiologically relevant environment in 3D, the spatiotemporal signalling pathways between cells may be investigated more accurately.

#### 2.1.1 Intra-tissue (Intercellular) communication

Organotypic skin models are a useful model to study tissue mechanisms and communication due to the high reproducibility of the model, and distinct, inducible changes in phenotype. Skin models are therefore often used to study ageing and disease, and the influence of senescent cells within a 3D environment has been primarily explored using living skin equivalents (LSEs), which are comprised of a differentiated epidermis on a fibroblast-populated dermal equivalent. LSEs constructed with keratinocytes from aged donors have a disorganised epidermal phenotype, characteristic of ageing, and an increase in the expression of p16 compared to models with young keratinocytes (Adamus et al., 2014). Interestingly, the presence of recombinant p16 in young keratinocytes caused an increase in endogenous p16 expression indicating a positive feedback loop. Conversely, silencing p16 led to a loss of the ageing phenotype, further supporting a role for p16 in the process of skin ageing (Adamus et al., 2014).

These models have also been used to explore the crosstalk that occurs between different cell types. For example, fibroblasts induced into a senescent state *via* mitomycin-C have been shown to cause an ageing epidermal phenotype in LSEs, demonstrating that senescent cell types can influence the surrounding tissues (Diekmann et al., 2016). Indeed, melanocytes with shortened telomeres can trigger telomere damage and reduced proliferation in neighbouring keratinocytes (Victorelli et al., 2019).

#### 2.1.2 Inflammatory phenotypes

Tissue engineering allows the culture of multiple cell types in a tightly controlled environment, and has previously been used to study immune cells and their mechanisms of inflammation. Activated T-cells (CD4+ cells) in an LSE constructed with normal human keratinocytes can cause a psoriatic phenotype, including hyperproliferation, reflective of the inflammatory state of psoriatic skin (Chamcheu et al., 2019). This work also validated the ability of fisetin, a compound of interest to the senescence field due to its capacity to selectively kill senescent cells (Yousefzadeh et al., 2018), to treat psoriatic symptoms by suppressing the inflammatory response (Chamcheu et al., 2019). Local inflammation in LSEs has been demonstrated by exposure to histamine, which caused a loss of keratinocyte differentiation markers and tight junction proteins, suggesting a mechanism by which histamine may contribute to inflammatory skin disorders by causing a defective skin barrier (Gschwandtner et al., 2013).

Microfluidic techniques have facilitated the investigation of circulating inflammatory signals on a multi-cellular tissue. A triculture model of Alzheimer’s disease (AD) using neurons, astrocytes and microglia within a microfluidic chamber formed physiologically relevant brain-tissue structures and more closely represented the secretome associated with AD when compared to 2D methods. The chemokine, CCL2 (C-C motif chemokine ligand 2), which has been shown to be upregulated in human AD brains, caused an increase in migratory and inflammatory phenotypes in microglia and T cells within the 3D model, and highlighted the relevance of such disease models in the study of inflammatory signalling pathways (Park et al., 2018).

Previous findings that disruption of epithelial homeostasis caused increased immune cell activation in the lung (Nguyen Hoang et al., 2012) led to further investigation into the effect of inflammatory signals in 3D lung models. Implantation of dendritic cells into a 3D human lung mucosa model revealed previously unobserved secretion of a number of CCL chemokine family members by dendritic cells (Nguyen Hoang et al., 2012), thus providing a platform to study immune regulation by the lung microenvironment. Multicellular 3D models such as this could be adapted to provide insights into the impact of senescent cells within a tissue on the wider immune system. These models also provide exciting opportunities to further understand the consequences of the SASP and senescent cells within a tissue-like environment. Insights from these strategies could then be applied to *in vivo* studies.

#### 2.1.3 Mechanisms

The mechanisms by which cells communicate within a 3D environment, both intracellularly and intercellularly, are still being elucidated. Organotypic models composed of cells surrounded by ECM are providing important insights. For example, conditioned media from human mammary fibroblasts (HMFs) cultured in 3D has increased levels of factors including interleukin-6 (IL-6), fibroblast growth factor 2 (FGF2) and hepatocyte growth factor (HGF). Furthermore, 3D conditioned media from HMFs caused non-invasive breast cancer cells to progress to an invasive phenotype, highlighting the importance of cellular crosstalk (Sung et al., 2013). Also of interest, 3D LSEs have been used to demonstrate fibroblast to keratinocyte crosstalk. In this model, fibroblast derived miRNAs from within the dermal compartment were detected within the epidermal compartment, *via* a process which may potentially be mediated by small extracellular vesicle delivery (Terlecki-Zaniewicz et al., 2019). This work, and that of others, suggests that cellular crosstalk may extend beyond soluble factors released into the extracellular milieu. Crosstalk between multiple cell types is extremely relevant within the senescence field as we consider the influence that a small senescent population may have on local neighbouring cells. The influence of the SASP on surrounding tissues and its global influence is still being uncovered, and may be investigated more closely though the use of organotypics.

### 2.2 Remodelling the microenvironment

Organotypic models are highly tuneable, and provide systems in which cells can be cultured with defined matrix composition and/or mechanical properties which can be modulated in order to investigate the relationship between cells and the underlying ECM. The effect of senescent fibroblasts on the surrounding ECM has been shown in a 3D *in vitro* dermal reconstitution system implanted onto the backs of severe combined immunodeficient (SCID) mice. In this system, late passage human dermal fibroblasts expressed reduced levels of collagen I and III, as well as increased levels of proteases associated with breakdown of the dermal matrix, including plasminogen activator, urokinase (uPA) and cathepsin O, compared to early passage fibroblasts (Funk et al., 2000). Importantly, the loss of these core ECM proteins resulted in the significant weakening of the structural integrity of the dermal matrix, consistent with the degeneration of dermal matrix architecture observed with chronological ageing (West, 1994).

Age-associated progressive degeneration of the dermis also alters the underlying mechanical properties of the dermal microenvironment. For example, dermal fibroblast size decreases during ageing as a consequence of reduced mechanical tension and subsequent impaired fibroblast-ECM attachment (Fisher et al., 2009). Using 3D collagen matrices of varying levels of stiffness, experiments have shown that decreased dermal fibroblast size in response to reduced mechanical force is associated with the lowered production of ECM components (Quan et al., 2013a). Recent studies have begun to investigate the molecular signalling triggered in response to these mechanical alterations; for example, reduced mechanical force decreases ECM production *via* down-regulation of transforming growth factor β-type two receptor (TβRII) leading to decreased activation of TGF-β/Smad signalling (Fisher et al., 2016).

Interestingly, ECM production can be significantly stimulated within dermal equivalent cultures (3D collagen matrices embedded with dermal fibroblasts) by enhancing the structural support of the dermis with injectable cross-linked hyaluronic acid dermal filler. Enlarged fibroblasts adjacent to the injection site produced significantly increased levels of TβRII, type I procollagen, and connective tissue growth factor (CTGF/CCN2) (Quan et al., 2013b).Taken together, these studies demonstrate that the relationship between cells and age-related changes in the underlying ECM composition/mechanobiology can be investigated *via* modelling of the 3D microenvironment *in vitro*.

### 2.3 Drug discovery and development

Recent studies have revealed novel opportunities for the application of 3D assays in drug discovery and development that can be applied to senotherapeutics, including senostatics and senolytics. 3D models offer an attractive alternative to current 2D *in vitro* and *in vivo* assays as they provide a more *in vivo*-like context than 2D assays and eliminate species difference by enabling drug testing directly in a human model system. For example, in a 3D LSE, treatment with a jasmonic acid derivative, LR2412, rescued the loss of ECM proteins observed with ageing, including collagen IV, indicating that the compound possesses ‘anti-ageing’ properties (Tran et al., 2014). Plant extract 1201, which has senolytic properties, blocked the detrimental effects of the SASP from stress-induced premature senescence (SIPS) HDFs in a human LSE causing rescue of the senescent cell dependent reduction in epidermal thickness and impaired differentiation of keratinocytes (Lammermann et al., 2018). In a 3D epidermal equivalent consisting of keratinocytes and melanocytes, incorporation of UV irradiated SIPS melanocytes induced paracrine senescence in surrounding keratinocytes and contributed to epidermal atrophy (Victorelli et al., 2019). Elimination of senescent melanocytes with a senolytic treatment, ABT-737, prevented paracrine effects on neighbouring keratinocytes and subsequent epidermal atrophy. Furthermore, treatment with a senostatic, mitochondria-targeted antioxidant, MitoQ, prevented paracrine telomere damage induction by senescent melanocytes and rescued epidermal thickness (Victorelli et al., 2019). Studies have also extended beyond *in vitro* models of skin. For example, explants of human adipose tissue have been shown to contain naturally occurring senescent cells and treatment of these with a senolytic combination, dasatinib and quercetin (D + Q), for 48 h resulted in significantly fewer senescent cells (Xu et al., 2018). Building on these collective findings, 3D assays have been validated for drug discovery in other age-related disease contexts; for example, proof-of-principle compound screening has been performed in genetically engineered stem cell-derived neurospheroids with familial AD mutations (Jorfi et al., 2018). The importance of understanding drug response in 3D has been highlighted by the differential response of breast cancer cells to drugs in 3D compared to 2D (Sung et al., 2013). Collectively, these studies establish 3D organotypics as an emerging experimental tool for the discovery and development of senotherapeutics in senescence research.

## 3 Future directions

Currently, senescence research is predominantly conducted using 2D tissue culture and *in vivo* animal models. However, we suggest that further scope exists for the use of 3D organotypic models to investigate both the bright and dark side of senescence, as illustrated in Fig. 1. For example, stem-cell derived organoids could be used to explore the mechanisms of senescence in development and the ‘one-two punch’ model for cancer therapy could be investigated using cancer organoids. Decellularised tissues would enable investigation of the effect of senescent fibroblast ECM derived matrices on proliferating cells, whilst 3D bioprinting offers the opportunity to use precision addition of senescent cells to microenvironments in order to investigate their influence on neighbouring cells. In addition, the paracrine effect of senescent cells could be further examined using ‘on-a-chip’ microfluidic chambers. Together, these suggestions represent a few possible investigative avenues amongst a multitude of many potential applications.

## 4 Concluding remarks

Organotypic 3D models present a valuable tool for studying both beneficial and detrimental mechanisms of senescence in an *in vivo* like context. These physiologically relevant models have previously enabled investigation of the role for senescence in a variety of processes, including paracrine signalling within tissues, and interactions with the ECM and the mechanical microenvironment. In addition, these models offer exciting potential as powerful high-throughput platforms for senotherapeutic drug discovery and development. As 3D organotypic models have been widely used to study a multitude cellular processes in a variety of cell and tissue types, there are potentially unlimited applications for 3D models in senescence research.
